# Medical and Philosophical Intelligence

**Published:** 1816-10

**Authors:** 


### MEDICAL AND PHILOSOPHICAL INTELLIGENCE.

*To the Editors of the London Medical and Physical Journal.*

GENTLEMEN,

I WAS much pleased, whilst reading your Journal for last month, to see the manner in which you had so wisely inserted, in p. 234, the valuable extract from the "*Sketch of the Medical History of the British Armies in the Peninsula of Spain and Portugal, by Sir James Macgrigor*,"—the perusal of which I consider as highly requisite to the young practitioner in both the military and naval services. To the young naval surgeon I particularly recommend his attention, as he may rest assured that such cases will very frequently occur in the course of his practice, especially in that class of men-of-war called gun-brigs. Sailors will often come to the surgeon, apparently labouring under every symptom of "oppression and debility," with a pulse scarcely to be felt. On inquiring into their complaint, they will only say, they are so "weak they cannot stand;" and, on asking whether they had any difficulty of breathing, or pain in the breast? they will tell him that they had a little load at the breast, but that it was of no consequence. On opening one of the veins of the arm, and a few ounces of blood having made its exit through a large orifice, the pulse will instantaneously become very full, and then, and not till then, will there be felt a great difficulty of breathing, and distinct pain in the breast. I am, &c.

*Portsmouth; Sept.* 11, 1816. J. W.

---

It is often remarked, that, whilst the other professions are open to the highest honors of the state, physic has no prospect beyond a baronetcy. It is, therefore, pleasing when we find testimonies of gratitude and respect bestowed by our fellow citizens, who must be the best judges of our usefulness. For this reason, we insert the following, hoping it may prove an example to other districts, who may have reason to be well satisfied with the long residence of an army practitioner. Dr. Pitcairn is a descendant of the venerable Archibald, and the head of his clan.

*From the Dublin Correspondent, August* 22, 1816.

"At a numerous meeting of the inhabitants of Athlone and its vicinity, the Lord CASTLEMAINE in the chair, the following address was unanimously agreed on:—

"To JAMES PITCAIRN, M.D. &c. &c.

"*Tholsel of Athlone, 10th August,* 1816.

"Sir.—We, the inhabitants of Athlone and its vicinity, at the Tholsel assembled, beg leave to express our sincere regret on your appointment to another district, and your consequent removal from this town and neighbourhood.

"During a residence of twelve years amongst us, passed in the most friendly and agreeable intercourse, we witnessed your unremitting mitting assiduity and skill in the duties of your profession—and, with admiration and gratitude, your constant and benevolent attentions to the interests of the poor.

"Impressed with these sentiments, we cannot suffer you to depart from amongst us without assuring you, that wherever you may be placed, there you will be attended by our warmest wishes for your welfare, and our best thoughts will be constantly employed upon you.

"Permit us to offer you, as a small token of our regard, a piece of plate, value one hundred guineas, to commemorate your residence amongst us, the recollection of which will be deeply engraven upon our hearts; and we trust you are assured of our being, with the truest esteem, your affectionate friends,

"And humble servants,

"*The Inhabitants of Athlone and its Vicinity.*

"(Signed) CASTLEMAINE, Chairman."

*Answer.*

"*Cork, August* 15, 1816.

"My Lord.—I have the honor to acknowledge the receipt of your lordship's letter of the 11th, containing an Address from the Inhabitants of the Town and Vicinity of Athlone, couched in language that will ever make a deep impression on my heart, and overrating services which were dictated only by a wish to serve my poor neighbours, as far as my feeble powers would admit, during leisure from my official duties. However gratifying the task may have been to my feelings, it is doubly recompensed in those expressions of regard and affection of which you have done me the honor to be the organ, and which I assure your lordship are truly reciprocal.

"The splendid token of esteem, I shall ever preserve as the most valuable gift that could be bestowed, and hand it down to my children as a memorial of the happiest period of my life, which has been passed with such serenity of mind and more flattering attentions from a large circle of friends in Athlone and its vicinity, than any merits of my own could claim.

"With these impressions, therefore, I need not add how painful my separation has been from friends, whose hospitality and kindness I have for so many years experienced, and in whose welfare and happiness I shall always feel the deepest interest.

"I cannot omit this opportunity of thanking your lordship for the truly gratifying manner in which you have communicated the above Address; thus adding one more to the many proofs of regard which I have had the honor of receiving from yourself and family.

"Believe me, my lord, under all circumstances your lordship's very faithful and obliged servant,

"(Signed) J. PITCAIRN, D. Inspector of Hospitals."

"*To the Right Hon. the Lord Castlemaine,*
*Moydrum Castle, near Athlone.*"

The publication of the above is highly proper, and makes a part of of the respect paid to the individual. For this reason we take the present opportunity of announcing a similar present made to one of our correspondents, but hitherto, we believe, not publicly announced. The following is the inscription on the cup.

Presented
to
JOHN TAUNTON, Esq.
by a few friends, subscribers to the
City of London Truss Society,
as a small testimony of their
Gratitude, Esteem, and Admiration,
excited by his
Disinterested Zeal, Unexampled Perseverance,
and Extraordinary Professional Abilities,
in Devising the Plan,
Increasing the Finances,
and Relieving the Objects,
of this
Excellent Institution.
1813.

---

The following passage is from Dr. Ashburner's inaugural Thesis. As these productions have only a limited circulation, we have thought it right to make the record more public.

"Huzard in dissertatione suâ, quam paucos abhinc annos coram Institutione Gallica legit, salivam quadrupedum herbâ vescentium malum afferre posse, negat. Amicus meus Dom. King, de Clifton, salivam bovi detractam, quæ rabie perierat, gallinæ inseruit. Post dies septuaginta et quinque, gallina, morbo affecta, periit. Doctor Macartney istam gallinam dissecuit; et, ubicunque inoculatione cutis incisa fuit, quendam tumorem parvum durumque in ejus superficie interiore invenit, magnitudine atque specie piso compresso haud absimilem, qui perquam esset vascularis."

---

LOBSTEIN'S *recent Remarks and Observations upon the Healing Power of Phosphorus.* Communicated by Dr. VON EMBDEN, at Hamburgh.

Professor LOBSTEIN, at Strasburgh, in his monography on phosphorus, in addition to the copious historical notices respecting its invention, its physical and chemical nature, and the use hitherto made of it in cases of sickness, has also published the results of his own experience; of the latter, we shall offer a very short account. Phosphorus is like other bold remedies: an improper use, or a disproportionate dose, produces dangerous consequences; it is, therefore, of great importance for the practical physician before he makes use of it, to be exactly informed of the manner of its application, and the dose to be administered.

Mr. L. objects to all the present pharmaceutical forms of phosphorus, except its solution in sulphuric æther. Only in this form, its caustic property is changed into a reviving and analeptic substance.

stance. According to Lœbenstein Lœbel's proposal, he adds a small quantity of a distilled aromatic oil, whereby not only the efficacy of the remedy is improved, but it is also more easily preserved; the solution of phosphorus is thus complete. To prevent its decomposition, it is proper to give it in a little syrup, or on a lump of sugar.

With regard to the dose, he has been taught by experience that in most cases one grain of phosphorus in twenty-four hours is quite sufficient, and that we ought neither to be too timid nor too bold in its administration; however, it will be always useful to begin with a small dose, and to increase or lessen the same, according to the symptoms. As soon as the patient feels any inconvenience, perceives any heat in the stomach, or vomits, it must be set aside.

Respecting the rules of precaution necessary in its application, Mr. Lœbenstein Lœbel's remarks are fully confirmed. The phosphorus agrees better, and can be borne in a larger dose, when the air is dry and clear than in cold and rainy weather. It ought never to be taken on an empty stomach, but always an hour after the patient has taken some nourishment; salad and acid food, and drink in general, even beer, is improper. To quench the thirst, a mucous solution of salep, with sweet and generous wine, is the best beverage; the patient must also refrain from drinking immediately after having taken the phosphorus.

In acute diseases, when there is commonly but little appetite, broth with a little nutmeg, or vermicelli, sago, &c. may be taken; but, in chronic disorders, where digestion is not impaired, veal, beef, and mutton, either boiled or roasted, fowls, snipes, hares, as also light vegetables, such as carrots, French beans, &c. are a fit diet. Cabbage, turnips, onions, radishes, rape, cole, pease, &c. must be avoided, causing a sensation of fullness in the region of the stomach, together with anxiety, insupportable heat, and often vomiting and diarrhœa. The food must neither be taken too hot nor too cold, fruit and milk are prohibited.

In case the patient can go out, it is of the greatest importance to be on his guard against catching cold, which is apt to occasion vertigo, and diarrhœa, or a relapse. Convalescents should wear a flannel shirt next to the skin; warm baths agree also very well with persons taking phosphorus, particularly in disorders of the nervous system, but they must not stay above a quarter of an hour in it.

*Effects of the Phosphorus upon the Animal Organism.*—In acute diseases, such as typhus, the effects of phosphorus appear frequently after four hours, but sometimes not till twenty-four. The vital warmth returns, transpiration is restored, the pulse improves, the urine is voided freely and commonly turbid, with a sediment; the abdomen loses its tension, the excrements have a sulphurious smell and shine in the dark; the delirium ceases, and the patient recovers his recollection, the mental faculties return, and a beneficial sleep restores the strength in a few days, the tongue grows clean and the appetite improves, and the countenance becomes chearful. These appearances, however, dont take place so so soon in persons advanced in years, they vary according to the nature of the disorder, and do not show themselves so distinctly in chronic cases.

Phosphorus is a remedy the effect of which extends over all the systems of animal economy, by rousing their activity, but it particularly operates upon the nervous system; the effect is very speedy and powerful, but of short duration. It is one of the most penetrating volatile excitants, and very particularly adapted for reviving the vital activity when nearly exhausted.

But this beneficial effect is only produced when the remedy is completely dissolved in its vehicle, when given in substance it operates as a violent caustic, equal to actual poison, by exciting most violent pains, burning, convulsion, tremor, annihilation of the powers, and death. At other times, if its use is too long continued, it causes the most obstinate complaints in the stomach; and, in case of a scirrhosity of the stomach, occasions death, as has been discovered afterwards on dissection.

Mr. L.'s own observations, communicated upon the efficacy of phosphorus in various disorders, are all of that kind, where the complaint has resisted other powerful remedies, or had been of long standing.

In three cases, where typhous persons were in the last stage of exhaustion, and where other irritative remedies seemed quite ineffectual; the vital activity was again roused in an astonishing manner by a few doses. The saving of a peripneumonic patient in the last stage of debility, performed by it, was equally unexpected. A tertian fever, obstinately resisting a variety of remedies, such as bark with flor. sal. amon. mart., opium with cinnamon, and even arsenic, was considerably lessened in the next attacks, and disappeared after phosphorus had been administered for eight days, during the intervals. A periodical cephalalgia in a very irritable woman, an obstinate cardialgia, and a chronic gouty complaint, wandering about in the body, was cured by it; menstruation, suppressed by catching cold, was restored, and the chlorotic patient recovered by the use of this remedy, combined with the tincture of cinnamon. It is superfluous to mention, that phosphorus, in the hands of a prudent physician, is a very powerful remedy, where the object is vigorously to rouse the vital activity, and to operate in a decisive manner; and Mr. L., by communicating these observations, in confirmation of the healing powers of this important remedy has acquired a decisive merit.

---

We are glad to find our neighbours begin to follow our illustrious countryman, though certainly *haud passibus æquis.* They see more and more the necessity of dismissing from physiology all the properties of chemistry, and every other consideration unconnected with the powers of life, or, as Mr. Hunter, on these occasions, called it, the *action of vessels.* Their language is, at present, a little too poetical, but it must lead them by degrees to sober reasoning; and, whenever this shall be the case, they will be able to comprehend the English physiologist by falling, it matters not by what means, into the same track. We are led to these reflections by the following communication from our correspondent at Paris:—

*Medical Bibliography for* 1816.—The formation of the pulmonary water, which presents insuperable difficulties to chemistry, appears under a very different aspect, when considered physiologically. It is sufficient to consider the water in vapour, which makes a part of the expired pulmonary gas, as the product of a real perspiration, or exhalation, analogous to that which takes place in the abdomen, in the breast, in the pericardium, in order to lubricate like a beneficent dew the surfaces contiguous to each other, to maintain the different organs in a state of suppleness and humidity necessary to the play of the parts, and to preserve the integrity of their functions.

I do not pretend to justify the too mechanical ideas of the immortal Boerhaave on Secretions; but, is it necessary equally to reject the plausible theory of Borden? This physiologist, full of sagacity, recognizes in each gland a kind of sentiment and taste, which does not permit it to receive into its tubes but one species of humour, to the exclusion of all others. He places secretion under the immediate influence of a particular nervous force, which makes the glandular organs experience titillations, and determines in the greater part of them an absolute erection. The inexorable reviser thinks Borden does not explain himself in a manner sufficiently clear and positive; I should be, I confess, less rigid than the author on this point, but I agree with him, that the chemists have not thrown any light on the nature of the prolific fluid. "That fluid so remarkable amongst the other wonders of organization for the property it possesses of communicating to germs by simple contact the first vital impulse; for I do not imagine that any physiologist, or physician, however he may have been led away by the seductions of modern chemistry, can ever forget himself so far as not to recognize the impenetrable obscurity which surrounds the mystery of the reproduction of beings, and to admit an explanation in the bottom of a crucible."

Assimilation is the result and consequence of nutrition, on which M. Cautanceau observes, the chemists ought to be silent: "They do not fail (says he) to insist on the notable quantity of phosphate of lime contained in milk, and in which they discover, as a final cause, the ossification of the new-born infant. I confess, I only see in it a barren physiological fact. Phosphate of lime appears to me to exist in milk only, because it is found in all albuminous liquids, without my being any better acquainted with the reason of it. Muriat of potass is equally met with, and in a still greater quantity; let them tell me then, why it is so, and for what purpose? for, from the presence of a calcareous phosphate in the first aliment of mammiferæ, serving to enlighten me on the mechanism of ossification, if I attached to the circumstance an importance that it does not possess, I should not have the less difficulty in conceiving how this function can be operated in the fœtus, deprived prived of such a resource; how it should continue in the infant after lactation, whatever species of food may be taken; and, how it happens to exist in reptiles and birds, who, in the first periods of their existence, are not fed with milk like the mammiferæ."

There are, however, certain phenomena really chemical, as pointed out by M. Cautanceau, for instance:—

1. The colouring of the skin and hair, by a long exposure to the action of the solar rays.

2. The coagulation by the mere contact of air of the albuminous matter, exuding from wounds and ulcers, and that of the blood itself, which becomes clotted at the extremity of the small vessels, divided by a recent wound.

3. The existence of soda in the state of *carbonate* in the mucous humour of the nostrils, while it presents itself in the state of caustic soda in all the other albuminous liquors in which it is met with.

4. The formation of concretions of various natures observed in different parts of the animal economy, and particularly the urinary calculi.

5. The action of several venomous substances, whether in the internal membrane of the stomach, or on each other.

6. The alteration, the acidulation, and coagulation of milk, and the oxidation of metals, introduced into the alimentary canal.

These are but trifling concessions; and M. Cautanceau evidently makes even these with regret, and endeavours to diminish the supposed importance of chemical applications, which are confined, he observes, to surfaces.

"I call *exterior*, with regard to the animal economy, (says this writer) the phenomena which actually pass within, and nothing can be more accurate than this definition for the liquids contained in the grand cavities of the human trunk, which receive the substances destined for absorption or secretion. As the stomach, the intestinal canal, the urinary bladder, and in particular the bronchial cavities, are evidently susceptible of being placed in immediate relation with the atmosphere, and to communicate with it without the aid of any other organ or faculty. Suppose the natural orifices sufficiently dilated, and the external air will penetrate of itself as easily all these depths as it touches the external surface of the skin. The fluids contained for the moment in these grand reservoirs may, therefore, be considered in idea as out of the body, or, more accurately, outside of the animal tissue which compose it."

☞ We shall select more on these subjects, and hope hereafter to find them more intelligible on the chemical coagulation of the blood, and on the chemical action of *venemous substances.*—By these, do they mean the chemical caustics?—Edit.

---

*Materia Medica of the Galibis and Guaripons, or Natives of Guiana.* From an unpublished work on the Colonies, by M. C. L. Cadet, (from the same source).

Medicine, amongst savage nations, is little more than quackery, rendered rendered worse by superstition and prejudice. It is however, to this source we owe the most active and the sole specifics on which we can bestow any degree of confidence: it is, therefore, interesting to study this gross system, if system it can be called, of therapeutics, giving it simply the degree of method requisite in the choice or classification of the materia medica.

Guiana produces a great variety of vegetables of use in medicine. The first Europeans established at Cayenne owed to the Gualipis, or Guaripons, the remedies they used before they transplanted their exotic medical plants. Aublet has given a botanical description of the vegetables of Guiana, and has traced some of their properties; but he has omitted:— we will endeavour to supply this omission; and, to render the matter clearer, we will present the plants, not in their botanical order, but according to their medical properties.

Amongst the vegetables produced by Guiana, we may enumerate nearly two hundred used in medicine, of which the most remarkable are the following:

PURGATIVES and EMETICS.

*Aloe perfoliata.*—The saccharine juice of this plant is laxative, vermifuge, and vulnerary. It is employed externally in dressing ulcers and gangrenes. There is also extracted from this aloe a nourishing fecula.

*Ambelania acida.*—The skin covering the fruit is purgative. The Galibis call it Ambelani and Paraveris.

*Argemone Mexicana.*—The seeds produce gentle evacuation, and are employed in dysenteries.

*Bignona copaia.*—This tree, called by the natives Pian ointment, is similar to the Simarbauba for its medical properties. Its bark is purgative, and sometimes emetic: they make a beverage of it, which is administered with success in diarrhœa and dysentery.

*Cassia fistula.*—Aublet observes that Cayenne produces thirteen species of Cassia. These trees are yet to be studied, as to the medical virtues of their fruit and leaves. It is one of the species of Cassia which produces those follicles which are sold as senna.

*Convolvolus Macrorhizos.*—The flower of this bind-weed is a purple bell, as large as the palm of the hand. Its root is very analogous to jalap, possesses its properties, and deserves to be studied as to its precise value in medicine.

*Gentiana exaltata.*—Its root is tonic, vermifuge, and purgative.

*Gratiola.*—Aublet announces this plant as coming from Virginia: he does not detail its properties. Is it bitter and purgative, like the European? This remains to be ascertained.

*Hernandia.*—With the kernels of the fruit of this tree, a purgative emulsion is formed. The inhabitants of Cayenne call these fruits Myrobolans.

*Hura crepitans.*—The Galibis call it Maman cacoa, and the inhabitants of Cayenne, Purge-parrot almonds. The emulsion is a violent violent purgative, which nearly killed several negroes to whom it had been administered. Its medical properties have not been properly attended to.

*Hypericum sessilifolium.*—The inhabitants call this species of St. John's wort Baptist-wood, blood-wood, fever-wood, &c. The resinous juice of the tree is administered in doses of seven or eight grains as a purgative.

*Medicago arborea.*—It is the same as that celebrated by Virgil as the Cytisa. The infusion of the leaves is purgative.

*Mirabilis.*—The root of this plant has been improperly considered as a true jalap. It is generally employed only in the veterinary art.

*Orelia allamanda.*—The decoction of this plant is a violent vomitive and purgative. Doctor Allamand employed it with success in the cholic of painters.

*Phylotacca.*—The berries, infused in brandy, used in frictions, give great immediate relief in rheumatism: taken internally, they purge; and are celebrated for their use in cancerous disorders.

*Piper Trifolium.*—The Spaniards and Portuguese use the leaves as tea: the infusion is slightly purgative.

*Painciana.*—Called by the inhabitants Peacock Flower and Flower of Paradise. It possesses similar virtues with senna.

*Polipody.*—Aublet found eight species in Guiana.

*Tamarinus Indica.*—The nature of the tamarind is well known.

*Verbena.*—Of the six species of Vervain described by Aublet one is purgative.

*Boerhavia.*—The root is emetic and purgative. The inhabitants call it, by analogy, ipecacuanha. Plumier and others have classed this plant, wrongly, under the Valerians: the Valerians are of the order Triandria, while the Boerhaave plant has only two stamens. If the properties be analogous to ipecacuanha, it would be interesting to make a comparative experiment of their merits.

*Cynanchum.*—Aublet has omitted to describe with precision the species that grows at Guiana. It requires to be examined whether it is the same species as that of Carolina, with the venomous juice of which the natives poison their arrows; or that called the ipecacuanha of the Isle of France, or the aromatic kind of Cochin-China.

*Viola calceolaria.*—The root possesses the properties of the white ipecacuanha, but it is much weaker.

*Cephœlio, tapogomea violacea.*—It is the true ipecacuanha of Brasil: five varieties are found in Guiana. It would be worth while to study them, and cultivate the most powerful.

FEBRIFUGES and ASTRINGENTS.

*The odorous Aristolochy.*—Its decoction, employed as a febrifuge, is equally used in lymphatic and glandular obstructions. It would be well to examine if, among the species, there be not the Serpentaria of Virginia.

*Cantarea.*—The bark of this tree, taken in decoction or in powder, is useful in intermittent fevers.

*Cascarilla.*

*Cascarilla.*—An excellent febrifuge. The bark precipitates solutions of iron of a beautiful black.

*Eryngium Fœtidum.*—The inhabitants of Cayenne consider it as an excellent specific in certain fevers.

*Exacum.*—A very bitter plant, used like common gentian.

*Fevillia.*—Called by the inhabitants Serpent-nut.

*Lecythis omara.*—This plant is not employed in medicine; but it is to be presumed, by analogy, that it possesses febrifuge and tonic properties. It would be well to make proper experiments with it.

*Lysianthus.*—This plant is of the gentian family, bitter and aperient. The Galibis employ it with effect in obstructions and fevers, particularly the kinds called *winged* and *purple.*

*Maranta arundinacea.*—Called by the natives Arrow-plant. When the Galibis have an intermittent fever, they cure themselves in eating the root baked in the ashes.

*Parulea.*—The bark is employed like Quinquina.

*Parthenium.*—Quoted by Aublet as a febrifuge.

*Phyllanthus, niruri & urinaria.*—These two plants are febrifuge and diuretic. They are employed in decoction in the suppression of the menses, in dysentery, and convulsions of children.

*Poincillade.*—Its flowers in decoction, like tea, cures the quartan fever.

*Simaboura amara.*—This vegetable is well known, but not much employed.

*Achyrantes.*—There are two species at Guiana, and both are astringent: they are used to stop the progress of the fluor albus, to cure ophthalmia in the commencement, inflammations, ulcers, slow fevers, and nocturnal perspiration. It would be well to examine if the Achyrantes of Guiana resemble those of India and China perfectly.

*Ambelania acida.*—Preserves, made of this fruit, are of use in dysenteries.

*Chrysobolanus icaco.*—The roots are very astringent.

*Coccolabo.*—There are two species at Cayenne. The kernel of the fruit is astringent. The natives use it against the dysentery.

*Costus Arabicus.*—The inhabitants eat the green stalks to stop a gonorrhœa.

*Epidendrum.*—There are twenty-three species at Guiana. It would be well to examine what relations they bear amongst themselves, and with the kinds used in medicine.

*Malpighia Moureila.*—The bark is used, in infusion, to stop looseness; it is said also to be febrifuge.

*Typha.*—An infusion of the roots is useful in involuntary loss of urine, dysentery, fluor albus, gonorrhœa, &c.

Vulnerary and Balsamic.

*The Oloe.*—Vide Supra, first article.

*Andropagon Insulare.*—This plant is detersive and vulnerary.

*Annona.*—The bark is pungent and aromatic. The Galibis use it in inveterate ulcers.

*Bacopa.*

*Bacopa.*—The inhabitants of Cayenne call this Burn-weed, because they suppose a cataplasm of it cures burns immediately.

*Bursera gummifera.*—It is called in St. Domingo Mountain Sugar Tree or Pig-wood. The tree has great analogy with that which produces gum arabic; is regarded as vulnerary.

*Clutia rosea.*—It is full of a milky viscous matter, which becomes red on exposure to the air. It is used for dressing the wounds of horses. It is called at St. Domingo the Cursed Fig-tree.

*Copaifera officinalis.*—It is the tree producing the Balsam of Capivi. As this balsam is generally had from Brazil, it would be well to examine their comparative merits.

*Myradendron balsamifera.*—This tree, cut, distils a balsamic liquor, like the Balsam of Peru; which dried, becomes a red, transparent, brittle resin, and burns with the odour of Gum Benzoin. A vulnerary spirituous liquor has been prepared from it, and the bark is used to burn as torches.

*Hydrocotyle umbellata.*—The roots are aromatic, vulnerary, and strongly aperient.

*Icica.*—A balsamic tree. Three species have been found at Guiana: they are called the Incense-tree.

*Arouaou Araconchini & Chipa.*—The resinous juice, extracted by incision, has a balsamic citron smell: it is burnt as a perfume, and the Galibis use it to heal wounds.

*Malpighia Verboscifolia.*—The decoction of the roots is used to cleanse ulcers. It is vulnerary and astringent.

*Matourea Vandelia.*—A plant called by the Creoles the Wild basilisk.

*Melastoma.*—There are twenty-one species at Guiana: they possess different degrees of virtue, and, in some respects, different qualities.

*Momordica balsamica.*—An excellent vulnerary. The fruit, infused in olive oil, is excellent for hæmorrhoides, burns, ulcers, &c.

*Ocymum.*—There are four varieties of this plant at Guiana.

*Omphalia diandra.*—The leaves, in decoction, are used to cleanse wounds and old ulcers.

*Origanum majorana.*—It is more active than in Europe.

*Paullinia pinnata.*—The fresh leaves are an excellent vulnerary.

*Thymus.*—More aromatic than in Europe.

*Urena.*—The root is considered as a specific against the sting of serpents.

*Varronea.*—Called by the inhabitants Mountain Sage. Is cephalic and astringent; and good in disorders of the bladder.

*Verbena.*—There are six species of Vervain found in Guiana.

---

Our correspondent from Germany has given an account of Experiments on Syphilitic Pus, which shall appear in a future Number. The following we transcribe, because we conceive the class of diuretic remedies cannot be too much increased.

Dr. Rehmann has, from his own observations, convinced himself of the efficacy of a remedy in dropsy, used as a house medicine by the peasants of Siberia. It consists in the herb of the Ballota lunata, growing in Siberia, from Teniscy to the Angara, on the dry declivities of the mountains, and on the other side of the Baikal, particularly between Werchney-Udinsk and Nert-shinsk. The plant is gathered at the latter end of the summer, when it has gone to seed; and is given with stalks and leaves together. Dr. R. uses it in the following manner:—One ounce and a half of the herb, grossly powdered, are boiled down with two pound of water to sixteen ounces and a half of tincture of cinnamon or orange, or, according to circumstances, ʒj. of simple or compound æther, and sometimes 15 to 20 drops of laudanum are added, and a cup-full taken every two hours.

The renal secretion commonly begins to increase after this remedy has been used from three to five days. The urine, which is white in the beginning, grows gradually darker, so that, by the sixth, seventh, or eighth day, it becomes almost quite black, or at least as dark as strong beer, being coloured by Ballota; and the less is left of it to be expelled, the darker does it grow. In many cases, a painful sensation in the hypochondria occurred when the dropsical swelling had nearly abated, which was always a sign that the use of the remedy could be gradually discontinued. It was now given in smaller doses, and in combination with bark and other corroborants, and was taken, as tea, night and morning, for some time longer.

In most cases, where the dropsy did not arise from organic mischief, the Ballota was found of the greatest service.

---

*Remarkable Instance of Sympathy of the Eye with the Intestines.*—Dr. Suck, of Wolmar, observed the following remarkable instance of retroversion of the pupil of the eye, from verminous irritation. A country girl, twelve years of age, was seized with a terrible head-ache, seated as well in the forehead, above the eyes, as in the occiput, signalised by a sensation of tearing and extension, hourly increasing in such a manner, that the patient, after five hours with dreadful furious looks, fell into a delirium furiosum, and shortly after, with a few convulsions, apparently expired. The relations, considering her as dead, treated her like a corpse. After twenty-four hours, however, she awoke again, and felt no more pain; but the orbits were filled up like with red flesh, and the optic foramen had disappeared. The bulb had been turned upwards in such a manner, that the part which, at other times, being turned in front, rests on the orbit with its muscles, appeared between the eye-lids, but the cornea and pupil were hidden under the upper part of the orbit. By help of the fingers, one could easily turn the eye downwards; but, letting it go, it immediately snapped back into its former situation. No disturbance of the functions was perceivable. The patient's age, and the peculiarity of the disorder, gave rise to the suspicion of worms. Anthelmintics and cathartics being given for three days, she passed passed ascarides, the same on the fourth day; the eyes now began to tremble convulsively, and a narrow edge of the white became visible. On the sixth day she passed a stool, consisting almost entirely of worms, whereupon directly the eyes resumed their natural position, and vision was restored.

---

We are honoured with a few other particulars of the late Dr. Squire, which we add with pleasure to the account of our last month.—Twenty-eight years ago, in conjunction with his friend, Mr. Chamberlaine, in whom he found a most ready and indefatigable coadjutor, the three branches of the medical profession were indebted to this worthy and excellent veteran for setting on foot an institution, not before attempted in the metropolis, for the benefit of the relatives of members of the medical profession left in distressed circumstances, under the title of *the Society for Relief of Widows and Orphans of Medical Men in London and its Vicinity.* By the unwearied exertions of these two individuals, this institution, from a small beginning, has arisen to a state of prosperity that its most sanguine supporters could not have expected in so short a period, and has afforded relief to many families, who, but for its assistance in many instances, must have endured the humiliating succours of a parish work-house.

---

Dr. Miller, late of Barbadoes, has succeeded to the residence and practice of Dr. Uwins in Aylesbury.

---

*Theatre of Anatomy,* 87, *Hatton Garden.* — The Autumnal Course of Lectures on Anatomy, Physiology, Pathology, and Surgery; by Mr. John Taunton, F. A. S. will commence on Saturday, October 5th, 1816, at eight o'clock in the evening precisely, and be continued every Tuesday, Thursday, and Saturday, at the same hour.

Dr. Adams's Course of Lectures begins on the 8th instant.

*Theatre of Midwifery, Berner's-street, Oxford-street.* — Dr. Clough, Physician-Accoucheur to the St. Mary-le-bone General Dispensary, and to the Endeavours, or Benevolent Society, for delivering Poor Women at their own Habitations, &c. &c. commences his autumnal Course of Lectures on the Science and Practice of Midwifery, including the Diseases of Women and Children, on Monday morning, the 7th of October, at half past ten, and his evening Course at seven.

Dr. Davis will commence his Winter Course of Lectures on the Theory and Practice of Midwifery, and on the Diseases of Women and Children, on Thursday, the 3d of October, at six o'clock in the evening, at Mr. Taunton's Theatre, No. 87, Hatton Garden.

Dr. Merriman and Dr. Ley will deliver a Course of Lectures on Midwifery and the Diseases of Women and Children, at the Middlesex Hospital, during the autumn. The introductory Lecture will be read on Monday, October 14, at half-past ten o'clock.

 Mr.

Mr. Guthrie, Deputy Inspector of Hospitals, will commence a Course of Lectures on the Principles and Practice of Medical and Operative Surgery, on the first Wednesday in October, at nine o'clock in the morning, at his house, No. 2, Berkeley-street, Berkeley-square.—To be continued Mondays, Wednesdays, and Fridays. Two Courses will be delivered during the season. The Operations in Surgery referred to in the Lectures will be shewn at the York Hospital, Chelsea, on the Friday mornings, and the treatment illustrated by such cases as may be in the hospital.

---

Mr. Salisbury's *Botanical Excursions, September* 1816.

September 3d.—Battersea Fields and Wandsworth Common.

Panicum viride.
——— crus Galli.
——— verticillatum.
——— dactylon.
Sagittaria sagittifolia.
Veronica scutellata.
——— Anagallis.
Cerastium aquaticum.
Melissa Nepeta.
Origanum vulgare.
Poa aquatica.
Polygonum Fugopyrum.
Pteris aquilina.
Scirpus acicularis.
——— cæspitosus.
Sison Amomum.
Senecio sarracenicus.
Stachys germanica.
Stachys palustris.
——— arvensis.
Solidago Virga-aurea.
Leonurus Cardiaca.
Bidens cernua.
——— tripartita.
Cyperus longus.
Carex incurva.
Centaurea Calcitrapa.
Datura Stramonium.
Erica multiflora (gardens).
——— Daboecia (ditto).
Inula Helenium.
——— dysenterica.
——— crithmoides.
Lysimachia vulgaris.
Antirrhinum elatine.
Arundo Calamagrostis.

September 12th.—North-fleet and Gravesend.

Aster Tripolium.
Matricaria maritima.
Atriplex pedunculata.
Artemisia maritima.
Chlora perfoliata.
Arenaria peploides.
Salicornia repens.
——— herbacea.
Asplenium Ruta-muraria.
——— Adiantum-nigrum.
——— lanceolatum.
——— Scolopendrium.
——— Ceterach.
Antirrhinum monspesulanum.
Zesteria marina.
Agrostis litoralis.
Chenopodium maritimum.
Dipsacus pilosus.
Erigeron canadense.
Valeriana rubra.
Arundo Phragmites.
Crocus officinalis.
Ophrys spiralis.
Scilla autumnalis.
Scabiosa succisa.
Samolus velerandi.
Sison verticillatum (garden).
Conyza squarrosa.

September 24th.—Virginia Water and Bagshot Heath.

Polypodium vulgare.

Arbulus Unedo.

Myrica Gale.

Carex depauperata.

Colchicum autumnale.

Hedera Helix.

Polypodium fontanum.

——— Dryopteris.

——— Phegeopteris.

——— aculeatum.

Pilularia globifera.

Senecio paluolosa.

The

The season being nearly over, I am not certain whether I shall make any more Excursions this autumn; but hope to resume this pleasant task next April, at which time I anticipate the pleasure of comparing the different periods of blooming of plants,—this being, in my opinion, the most backward season that has ever been noticed, so much so, that there will scarcely be time for the acorns or chesnuts to grow to their usual and full size. Several of the gentlemen who have honoured me with their attendance this summer, and who are now settled in different parts of the country, have obligingly offered to compare notes, on this head, with my class next season,—whence I hope to give a new interest to my future communications.

Those who have honoured me with their attendance, will recollect that it is my principal object to teach, as far as possible, the *various uses* to which *each plant* is destined in the economy of life; and I am happy that so far my efforts have been successful in pointing out how a considerable saving may be made, by teaching the cottager and labouring classes of society how many plants, totally disregarded at this time, may become substitutes for others purchased at high prices from abroad, and at a season too when thousands are starving for want of employment.

These remarks will, no doubt, catch the eye of some of my fellow-labourers in botanic science, who have heard me regret that an establishment for promoting more particularly cottage and rural economy has not been made in this country; and they will also recollect that I have often remarked, when walking on the bank of the Thames, that the *Scirpus lacustris (bull-rush)*, which grows there so fine, and in such abundance, might some day become a source of wealth, if our attention were turned towards domestic improvement. I am now happy to observe that these remarks have not been useless. The quantity of the same rush imported last year (1815) from Holland was 149,000,273 bundles, which have been purchased at upwards of nine thousand pounds, *paid for in cash*, while we have acres of the same article, as well as a similar one, that is a better substitute for all purposes to which it is applied, growing to waste every year, which would find employment for thousands, and save our money at home. The remarks we have also occasionally made as to the plants used in dyeing (which are only in use by the country people), the collecting seeds of our best meadow grass, tares, &c. grown on waste lands, and also such plants as are productive of hemp and similar materials, may be turned to good account,* as I have often observed in the

* I have this autumn had a number of experiments made to prove these facts, and have by me the articles manufactured, which I have submitted to the inspection of several of our most eminent agriculturists, &c. who have been convinced of the utility of the object; and I shall at all times be happy to shew them to any persons who feel interested in the benefit of our labouring poor.

Botanist's

Botanist's Companion.* From that small work, with some further remarks communicated personally to the President of the Society of Arts, his Royal Highness was pleased to express his pleasure that an institution should be formed under the name of the Œconomic Garden; the object of which should be to display more generally all these advantages, and to disseminate such information among the poor living in the neighbourhood where those plants most abound, but which, through ignorance of their worth, are at present turned to no account whatever. This proposed institution is not sufficiently matured to give you the plan in detail; as, I trust, I shall be able to do in your next Number.

*Botanic Garden, Sloane-street;* I am, &c.
*Sept.* 26, 1816. W. Salisbury.

---

### REPORT OF DISEASES.

Inflammation is still the epidemic, chiefly in the larger cavities. The thorax, the heart, and its appendices, are almost as often affected as the pleura and lungs. The diaphragm has been marked out with peculiar exactness as the seat by a few patients. In others, both cavities seem to have been affected in succession. In all, the free use of the lancet has been, if not the *unicum*, at least the *remedium princeps;* and, where applied in time and with vigor, has, in every instance which has occurred to the Reporter, proved successful; at the same time it has been thought prudent to evacuate the bowels very freely, nor have we found that interruption from the difficulty of affecting them which often happens in inflammatory diseases.

A case has occurred so interesting, that we cannot sufficiently regret an examination was not permitted. The patient had been ill nearly a week, with pains about the abdomen which progressively encreased. He had been bled, but neither early nor freely; his bowels had been sufficiently operated upon. When the Reporter saw him (the night before death) the pain had nearly subsided, but the sickness was very considerable, and vomitings frequent; the tongue clean, the pulse below 80. The most alarming symptoms were a hurried respiration and universal coldness. These continued till the close.—Query, Was this the *sequela* of inflammation, or was there reason to suspect poison? The cleanness of the tongue and fauces make it difficult to discover any other satisfactory cause. The Reporter will be thankful for the opinion of any of his brethren to whom similar cases may have occurred.

Small-pox continues, we may say increases, its ravages. Is the inattention to the blessing of vaccination to be ascribed to an indifference to life, or to mere indolence? In the softer sex, we might suppose a regard to personal beauty might produce its effect; probably, it is for this reason that the sufferers are principally infants or adult males.

---

* A review of this useful little performance will appear in an early number.—Edit.

### METEOROLOGICAL REGISTER.

*From August the 25th, to September the 25th,* 1816.

Kept by C. BLUNT, Philosophical Instrument Maker, No. 38, Tavistock-Street, Covent-Garden.

| Moon. | Day. | Wind. | Barometrical Pressure. | | | Temperature. | | | |
|---|---|---|---|---|---|---|---|---|---|
| | | | Max. | Min. | Mean. | Max. | Min. | Mean. | |
| | 26 | NW | 30·30 | 30·25 | 30·27 | 61 | 40 | 50·5 | Showers |
| | 27 | NW | 30·21 | 30·20 | 30·20 | 60 | 44 | 52· | Fair |
| | 28 | NW | 30·24 | 30·23 | 30·23 | 63 | 44 | 53·5 | Fair |
| ◑ | 29 | NW | 30·26 | 30· | 30·13 | 60 | 43 | 51·5 | Fair |
| | 30 | NW | 29·50 | 29·30 | 29·40 | 60 | 40 | 50· | Rain |
| | 31 | NW | 29·40 | 29·27 | 29·30 | 56 | 39 | 47·5 | Rain |
| | 1 | N | 29·45 | 29·42 | 29·43 | 50 | 38 | 44· | Rain |
| | 2 | N | 29·42 | 29·36 | 29·39 | 52 | 32 | 42· | Rain |
| | 3 | N | 30· | 29·62 | 29·81 | 54 | 39 | 46·5 | Rain |
| | 4 | NW | 30· | 29·70 | 29·85 | 55 | 38 | 46·5 | Rn.& hail |
| | 5 | W | 29·69 | 29·67 | 29·68 | 59 | 42 | 50·5 | Rain |
| ○ | 6 | W | 29·66 | 29·65 | 29·65 | 61 | 44 | 52·5 | Fair |
| | 7 | W | 29·64 | 29·62 | 29·63 | 60 | 43 | 51·5 | Fair |
| | 8 | SW | 29·67 | 29·64 | 29·65 | 61 | 44 | 52·5 | Rain |
| | 9 | SW | 29·71 | 29·69 | 29·70 | 62 | 43 | 52·5 | Fair |
| | 10 | S | 29·89 | 29·82 | 29·85 | 63 | 42 | 52·5 | Fair |
| | 11 | SW | 30· | 30· | 30· | 65 | 45 | 54· | Fair |
| | 12 | W | 30·26 | 30·20 | 30·23 | 67 | 44 | 55·5 | Fair |
| | 13 | W | 30·34 | 30 | 30·17 | 69 | 43 | 57· | Fair |
| ◐ | 14 | S | 30· | 30· | 30· | 70 | 46 | 58· | Fair |
| | 15 | S | 30·2 | 30·10 | 30·15 | 80 | 53 | 66·5 | Fair |
| | 16 | S | 30·1 | 30· | 30·05 | 82 | 53 | 67·5 | Fair |
| | 17 | S | 29·86 | 29·85 | 29.85 | 76 | 49 | 63· | Fair |
| | 18 | SW | 29·84 | 29·75 | 29·79 | 70 | 46 | 58· | Fair |
| | 19 | SW | 29·93 | 29·70 | 29·86 | 66 | 47 | 56·5 | Fair |
| | 20 | W | 29·77 | 29·75 | 29·76 | 66 | 48 | 57· | Fair |
| ● | 21 | W | 29·73 | 29·70 | 29·71 | 63 | 46 | 54·5 | Rain |
| | 22 | W | 29·66 | 29·64 | 29·65 | 64 | 45 | 54·5 | Fair |
| | 23 | NW | 29·64 | 29·63 | 29·63 | 65 | 44 | 54·5 | Rain |
| | 24 | SW | 29·64 | 29·63 | 29·63 | 66 | 43 | 54·5 | Fair |
| | 25 | S | 29·92 | 29·75 | 29·88 | 66 | 43 | 54·5 | Fair |

RESULTS.

Mean barometrical pressure of the month — 29·82
Maximum 30·23, wind at NW
Minimum 29·30, —— NW

Mean temperature of the month — 54·5 deg.
Maximum 82, wind at S
Minimum 32, —— N

*Scale exhibiting the prevailing Winds during the Month.*

| N | NE | E | SE | S | SW | W | NW |
|---|---|---|---|---|---|---|---|
| 3 | 0 | 0 | 0 | 6 | 6 | 8 | 8 |

